# Dexmedetomidine-induced hemodynamic instability in patients undergoing orthopedic upper limb surgery under brachial plexus block: a retrospective study

**DOI:** 10.1186/s12871-021-01416-4

**Published:** 2021-09-16

**Authors:** A Ram Doo, Hyungseok Lee, Seon Ju Baek, Jeongwoo Lee

**Affiliations:** 1 Department of Anesthesiology and Pain Medicine, Jeonbuk National University Hospital and Medical School, 20 Geonji-ro, Deokjin-gu, Jeonju, 54907 Jeollabuk-do South Korea; 2 Research Institute of Clinical Medicine of Jeonbuk National University-Biomedical Research Institute of Jeonbuk National University Hospital, Jeonju, South Korea

**Keywords:** Brachial plexus block, Dexmedetomidine, Procedural sedation, Regional anesthesia, Hypotension, Obesity, Orthopedic, Perioperative

## Abstract

**Background:**

Hemodynamic instability is a frequent adverse effect following administration of dexmedetomidine (DMED). In this study, we evaluated the incidence of DMED-induced hemodynamic instability and its predictive factors in clinical regional anesthesia practice.

**Methods:**

One hundred sixteen patients who underwent orthopedic upper limb surgery under brachial plexus block with intravenous DMED administration were retrospectively identified. The primary outcome was the incidence of DMED-induced hemodynamic instability. The participants were allocated to a stable or unstable group by their hemodynamic instability status. Patients’ characteristics were compared between the groups. The relationship between the potential risk factors and development of DMED-induced hemodynamic instability was analyzed with a logistic regression model.

**Results:**

DMED-induced hemodynamic instability was observed in 14.7% of patients (17/116). The unstable group had more women than the stable group (76.5% vs. 39.4%, *P* = 0.010). When patients were classified into four subgroup according to body mass index (underweight, normal weight, overweight, and obesity), there was significant difference in the composition of the subgroups in the two groups (*P* = 0.008). In univariate analysis, female sex, obesity, and pre-existing hypertension were significant predictors of DMED-induced hemodynamic instability. Multivariate analysis demonstrated that female sex (adjusted OR 3.86, CI 1.09; 13.59, *P* = 0.036) and obesity (adjusted OR 6.41, CI 1.22; 33.57, *P* = 0.028) were independent predictors of DMED-induced hemodynamic instability.

**Conclusions:**

Female and obese patients are more likely to have hemodynamic instability following intravenous DMED administration in clinical regional anesthesia practice. This study suggests that DMED dose may be diminished to prevent hypotensive risk in these populations.

**Trial registration:**

This article was retrospectively registered at WHO clinical trial registry platform (Trial number: KCT0005977).

## Background

Regional anesthesia increasingly expands its role in perioperative care. The clinical benefits of regional anesthesia include better postoperative analgesia, preserved consciousness during surgery, and possibly lower incidence of postoperative delirium or cognitive dysfunction. However, in clinical practice, because awake patients often complain of anxiety or discomfort during surgical procedures regardless of the type of regional anesthesia provided, various sedatives and additional analgesics are commonly used. Dexmedetomidine (DMED), a highly selective α2-adrenergic agonist, is the most preferred sedative because of its advantages. One of these is conscious sedation with minimal respiratory depression, enabling the patients to be more cooperative during the intervention. DMED also manifests sympatholytic, sedative, hypnotic, amnesic, and analgesic properties. Consequently, DMED is increasingly used for procedural sedation during intervention, sedation in intensive care patients with mechanical ventilation, and as an adjuvant in balanced anesthesia. Particularly, the benefits of DMED, when combined with regional anesthesia, includes increasing the regional anesthesia quality, prolonging postoperative analgesia, and endowing an opioid-sparing effect [1–3].

It is well known that the pharmacologic effect of DMED in the cardiovascular system includes the reduction of blood pressure and heart rate in a dose-dependent manner by activating the peripheral α2-adrenoreceptor [4–6]. Indeed, the occurrence of hemodynamic instability after DMED administration, including hypotension or bradycardia, has been reported in several investigations. The reported incidence of hemodynamic instability in the intensive care units (ICU) ranges from 20.6–71% [7–10]. Several risk factors for DMED-induced hemodynamic instability, including older age and lower baseline blood pressure, were suggested [9]. However, these studies were limited to ICU settings. To the best of our knowledge, there had been no well-designed study to evaluate DMED-induced hemodynamic instability in clinical regional anesthesia practice, even though patients often experience such events in clinical practice.

In this study, we retrospectively evaluated the development of hemodynamic instabilities such as hypotension and bradycardia in patients administered with intravenous DMED for sedation during orthopedic upper limb surgery under brachial plexus block (BPB). The study aimed to evaluate the incidence of DMED-induced hemodynamic instabilities and determine predictive factors for such instabilities during procedural sedation in regional anesthesia practice.

## Methods

**Fig. 1:**
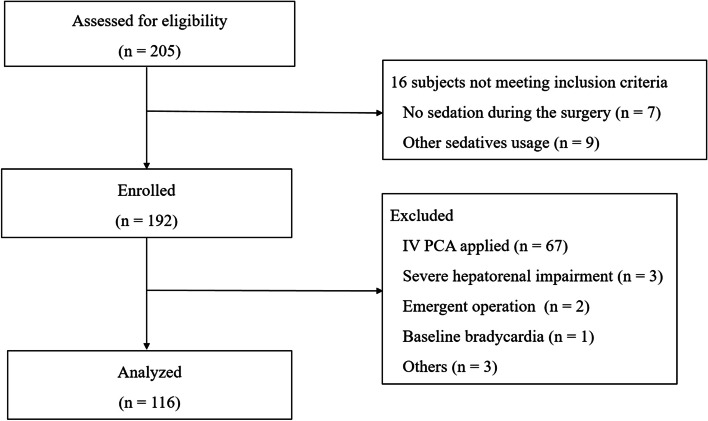
Subject flow diagram

This retrospective study was approved by the Institutional Review Board of Jeonbuk National University Hospital, Jeonju, South Korea, and the need to obtain informed consent was waived based on the Good Clinical Practice regulations and guidelines. This manuscript adheres to the applicable STROBE guidelines. We retrospectively evaluated the medical records of 205 consecutive patients who underwent orthopedic upper limb surgery under BPB at our institution between March 2017 and February 2020. Inclusion criteria were as follows: age $\ge$ 18 years, American Society of Anesthesiologists (ASA) physical status (PS) I-III, administered with intravenous DMED during the surgery. Among the 192 patients enrolled, we analyzed the data of 116 patients in this study. We excluded from the analysis the following patients: 1) Patients who were given an intravenous opioid-based patient-controlled analgesia device at the end of anesthesia (*n* = 67), 2) Patients who had severe hepatic or renal impairment (*n* = 3), 3) Emergent operation (*n* = 2), 4) Patients whose pre-anesthetic heart rate was less than 50 beats per minute (bpm) (baseline bradycardia; *n* = 1), and 5) Others (*n* = 3). Subject selection is presented as a flow diagram in Fig. 1.

The primary outcome was the incidence of DMED-induced hemodynamic instability. The participants were classified into the stable or unstable groups based on their hemodynamic instability status. Secondary outcome included the patients’ characteristics in each subgroup and the predictive risk factors associated with the development of DMED-induced hemodynamic instability. The patients’ demographic data, including age, sex, height, weight, body mass index (BMI), and general medical condition, were collected by reviewing the medical charts. The presence of underlying diseases such as hypertension, cardiovascular disease, cerebrovascular disease, and diabetes mellitus was also recorded. The data related to the DMED usage during the operation included total drug administered and the infusion time. Concomitantly administered drugs, such as benzodiazepine or fentanyl, were also recorded. In all patients, hemodynamic parameters, including blood pressure and heart rate, were assessed at specific time points from the start of DMED infusion until the patients were discharged from the postanesthetic care unit (PACU).

The development of DMED-induced hemodynamic instability, such as hypotension or bradycardia, was identified. Hypotension was defined as systolic blood pressure (SBP) decreased by more than 30% of the baseline and/or less than 90 mmHg, and bradycardia was defined as a heart rate of less than 50 bpm. To qualify as an event, SBP less than 90 mmHg had to be recorded for at least two consecutive readings at 10-min interval. The occurrence of these events was evaluated for five hours from the initiation of DMED infusion. Based on this evaluation, the participants were allocated to a stable or unstable group. The stable group included patients who did not experience hypotension and bradycardia, while the unstable group included patients presenting hemodynamic instability during the observation period. Meanwhile, transient hypertension following DMED loading was also identified. DMED-induced hypertension was defined as SBP increased by more than 30% of the baseline and/or more than 180 mmHg.

### Anesthesia management

The ultrasound-guided supraclavicular BPB was performed in all patients enrolled in the present study. After identifying the brachial plexus using a 13–6 MHz linear array transducer (EDGE® ultrasound machine, Sonosite Inc., USA), a 25-gauge, 5 cm block needle was inserted toward the brachial plexus with a lateral to medical direction. Then, half the volume (16 ml) of 1.5% lidocaine with epinephrine 5 μg/mL was injected into the main neural cluster. The remaining half (16 ml) was then injected into every satellite neural cluster by the previously described targeted intracluster injection method [11]. After a reliable motor and sensory block was confirmed, the infusion of DMED was initiated to achieve patients’ sedation.

At our institution, when sedation is required during surgical procedure under regional anesthesia, the intravenous DMED infusion protocol follows the manufacturer’s recommendation, which includes a standard initial loading dose of 1 μg/kg over ten minutes, and subsequent maintenance rate of 0.2–0.6 μg/kg/hr until the end of the surgery. The maintenance rate may be titrated to achieve the target Modified Observer’s Assessment of Alertness/Sedation scale 3–4, representing moderate sedation. On this scale, 5 = Responds readily to name spoken in a normal tone, 4 = Lethargic response to name spoken in a normal tone, 3 = Responds only after name is called loudly or repeatedly, 2 = Responds only after mild prodding or shaking, 1 = Responds only after painful trapezius squeeze, 0 = No response after painful trapezius squeeze.

### Statistical analysis

Statistical analysis was performed using IBM SPSS Statistics for Windows, version 25 (IBM Corp., Armonk, N.Y., USA). All descriptive data are expressed as mean (SD), median (interquartile range), and the number of patients (%).

We first compared the clinical characteristics, including demographic data and the data related to DMED usage, between the stable and unstable groups. Two-tailed independent-samples *t*-test or Mann–Whitney rank-sum *U* test was used to analyze continuous variables after performing Shapiro–Wilk test. Chi-square test was used to compare categorical variables. Hemodynamic parameters, such as blood pressure and heart rate in both groups, were analyzed with two-way repeated measures analysis of variance (RM ANOVA), and post-hoc analysis was performed by the Bonferroni correction procedure. Based on the statistical comparisons between the two groups, univariate logistic regression analysis was performed to identify potential risk factors for DMED-induced hemodynamic instability, with the crude odds ratios (ORs) and their 95% confidence intervals (CIs). The statistically significant variables in the univariate analysis were integrated into a multivariate logistic regression model, and the adjusted ORs, 95% CIs, and *p* values were calculated for each variable. After the logistic regression model was established, further analysis included Kaplan–Meier survival method to estimate the cumulative incidence of DMED-induced hemodynamic instability and log-rank test to compare the survival curves between stratified patients’ groups. Differences with a two-tailed *p* value of < 0.05 were considered statistically significant.

## Results

The DMED-induced hemodynamic instability was observed in 17 of the 116 patients (14.7%), of which 16 experienced hypotension, and one had bradycardia after DMED administration. These patients comprised the unstable group, while the rest (*n* = 99) formed the stable group. The patients’ characteristics were compared between the stable and unstable groups (Table 1). The median ages in the two groups were comparable. In the unstable group, the proportion of females was significantly higher than in the stable group (76.5 vs. 39.4%, *P* = 0.010). Even though the BMI in the two groups was similar by two-tailed independent-samples *t*-test, when the patients of both group were classified into four subgroups by the BMI (< 18.5 kg/m^2^, underweight; 18.5–24.9 kg/m^2^, normal weight; 25.0–29.9 kg/m^2^, overweight; $\ge$ 30.0 kg/m^2^, obese), there was significant difference in the composition of the subgroups between the stable and unstable groups (*P* = 0.008 by two-tailed Mann–Whitney rank-sum *U* test). Among the patient’s underlying medical conditions, hypertension was more prevalent in the unstable group than in the stable group (47.1 vs. 20.2%, *P* = 0.037 by Chi-square test). All patients with a history of hypertension, in both groups, were taking one or two anti-hypertensive medications, with no difference between the groups. The groups were also comparable in terms of the prevalence of coronary arterial disease and diabetes mellitus. Regardless of the development of DMED-induced hemodynamic instability, there was no difference between the groups in the DMED therapy characteristics, including the total consumption of the administered drug, total infusion time, or total drug administered per body weight (Table 2). The concomitant administration of other sedatives was also similar between the groups.

**Table 1:** Patient characteristics, stratified by hemodynamic instability status

	Stable group (*n* = 99)	Unstable group (*n* = 17)	*P* value
Age (years)	54.0 (36.0–62.0)	59.0 (48.0–68.0)	0.057
Female [n (%)]	39 (39.4)	13 (76.5)	0.010^*^
Body weight (kg)	65.6 (12.6)	63.2 (10.0)	0.449
BMI (kg/m^2^)	24.3 (3.6)	26.1 (4.0)	0.058
Classification by BMI			
Underweight [n (%)]	5 (5.1)	0	0.008^†^
Normal weight [n (%)]	52 (52.5)	5 (29.4)	
Overweight [n (%)]	38 (38.4)	8 (47.1)	
Obesity [n (%)]	4 (4.0)	4 (23.5)	
Underlying disease [n (%)]			
Hypertension	20 (20.2)	8 (47.1)	0.037^*^
History of CAD	4 (4.0)	1 (5.9)	0.764
Diabetes mellitus	12 (12.1)	3 (17.6)	0.813
Preoperative laboratory test			
AST (IU/L)	23.0 (20.0–28.0)	25.0 (20.5–34.0)	0.296
ALT (IU/L)	22.0 (18.0–29.0)	21.0 (19.0–38.0)	0.550
Albumin (g/dL)	4.6 (4.3–4.7)	4.4 (4.3–4.6)	0.094
Surgery time (min)	37.0 (25.0–56.0)	30.0 (25.0–53.5)	0.734
PACU stay (min)	62.0 (53.0–74.0)	57.0 (50.0–93.0)	0.896
Hospital stay (days)	6.0 (4.0–8.0)	6.0 (5.0–9.5)	0.195
Time to hemodynamic instability after initiating DMED administration (min)	N/A	67.0 (37.4–96.6)	N/A

Continuous variables are presented as mean (SD) or median (interquartile range). Categorical variables are presented as number (%)

Underweight < 18.5 kg/m^2^; normal weight 18.5–24.9 kg/m^2^; overweight 25.0–29.9 kg/m^2^; obesity $\ge$ 30.0 kg/m^2^

*BMI* body mass index, *CAD* coronary artery disease, *AST* aspartate aminotransferase, *ALT* alanine animotransferase, *PACU* postanesthesia care unit, *DMED* dexmedetomidine, *N/A* not applicable

^*^P < 0.05 by chi-square test

^†^*P* < 0.05 by Mann–Whitney rank-sum test

**Table 2:** Dexmedetomidine therapy characteristics

	Stable group (*n* = 99)	Unstable group (*n* = 17)	*P* value
Total consumption of DMED^a^ ($\upmu$ g)	78.1 (65.6–89.0)	78.4 (62.5–91.6)	0.746
Total infusion time of DMED (min)	40.0 (28.0–60.0)	40.0 (20.0–56.5)	0.673
Total consumption *per b.w.* ($\upmu$ g/kg)	1.2 (1.1–1.3)	1.2 (1.1–1.3)	0.894
Concomitantly administered sedatives [n (%)]			
Benzodiazepine	49 (49.5)	5 (29.4)	0.204
Fentanyl	11 (11.1)	1 (5.9)	0.824

Continuous variables are presented as median (interquartile range), and categorical variables are presented as number (%)

*DMED* dexmedetomidine, *b.w.* body weight

DMED-induced transient hypertension was observed in 6 of the 116 patients (5.2%), and all patients were allocated in the stable group. Among them, two had a history of concomitant hypertension and diabetes mellitus, two had a history of diabetes mellitus for over ten years, and one had a history of hypertension. The transient hypertension in all six patients was well controlled within a few minutes with intravenous anti-hypertensive drugs, without any significant complications.

**Fig. 2:**
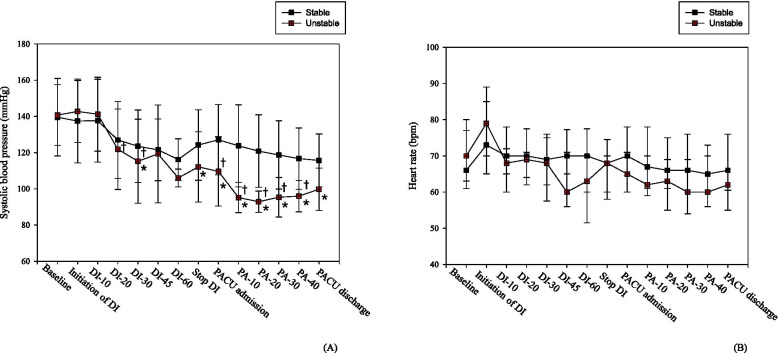
The changes of systolic blood pressure (**A**) and heart rate (**B**) in the stable and unstable groups. DI; Dexmedetomidine infusion, DI-10; 10 min after initiation of DMED infusion, DI-20; 20 min after initiation of DMED infusion, DI-30; 30 min after DMED infusion, DI-45; 45 min after DMED infusion, DI-60; 60 min after DMED infusion, PACU; postanesthesia care unit, PA-10; 10 min after PACU admission, PA-20; 20 min after PACU admission, PA-30; 30 min after PACU admission, PA-40; 40 min after PACU admission. ^*^ When compared to baseline, the systolic blood pressure was significantly lower at 30 min after DMED infusion (*p* = 0.013), when infusion was stopped (*p* = 0.014), at admission to the PACU (*p* < 0.001), 10 (*p* < 0.001), 20 (*p* < 0.001), 30 (*p* < 0.001), and 40 (*p* < 0.001) minutes after PACU admission, and on discharge from PACU (*p* < 0.001), respectively, in the unstable group. ^†^ The systolic blood pressures were significantly different at 20 min after DMED infusion, 30 min after DMED infusion, at the PACU admission, 10 min after PACU admission, 20 min after PACU admission, 30 min after PACU admission, and 40 min after PACU admission between the two groups. (*p* = 0.017, *p* = 0.024, *p* = 0.037, *p* = 0.001, *p* < 0.001, *p* = 0.022, and *p* = 0.013, respectively). DMED; dexmedetomidine

The changes in SBP and heart rate following DMED administration are presented in Fig. 2. Within the stable group, there was no difference in the parameters over time. Meanwhile, within the unstable group, the SBP tended to decrease continuously over time, and it did not recover to baseline until the patients were discharged from the PACU. When compared to baseline, the SBP was significantly lower at 30 min after DMED infusion (*P* = 0.013), when infusion was discontinued (*P* = 0.014), at admission to the PACU (*P* < 0.001), at 10, 20, 30, and 40 min after PACU admission (*P* < 0.001 for all), and on discharge from PACU (*P* < 0.001), by the two-way RM ANOVA. In the unstable group, the SBP had maximally decreased by 32.9% below the baseline at 20 min after PACU admission. Between the two group, the SBP was significantly different at 20 (*P* = 0.017) and 30 (*P* = 0.024) minutes after the start of DMED infusion, at the PACU admission (*P* = 0.037), and at 10 (*P* = 0.001), 20 (*P* < 0.001), 30 (*P* = 0.022), and 40 (*P* = 0.013) minutes after PACU admission. There was no difference in the heart rate, both within and between the groups.

The univariate analysis revealed that sex (female), obesity (BMI $\ge$ 30 kg/m^2^), and pre-existing hypertension were significant predictors of DMED-induced hemodynamic instability (Table 3). In the multivariate analysis, both female sex (adjusted OR: 3.86, 95% CI: 1.09–13.59, *P* = 0.036) and obesity (adjusted OR: 6.41, 95% CI: 1.22–33.57, *P* = 0.028) were found to be independent predictors of DMED-induced hemodynamic instability.

**Table 3:** Risk factors for dexmedetomidine-induced hemodynamic instability by logistic regression analysis

	Univariate analysis		Multivariate analysis	
	Crude OR (95% CI)	*P* value	Adjusted OR (95% CI)	*P* value
Female sex	5.00 (1.52–16.45)	0.008^*^	3.86 (1.09–13.59)	0.036^*^
Obesity	7.31 (1.63–32.82)	0.009^*^	6.41 (1.22–33.57)	0.028^*^
Concomitant HTN	3.51 (1.20–10.25)	0.022^*^	2.37 (0.73–7.71)	0.152

*OR* Odds ratio, *CI* confidence interval

Obesity corresponds to body mass index $\ge$ 30 kg/m^2^

*HTN* hypertension

^*^P < 0.05 was considered statistically significant

**Fig. 3:**
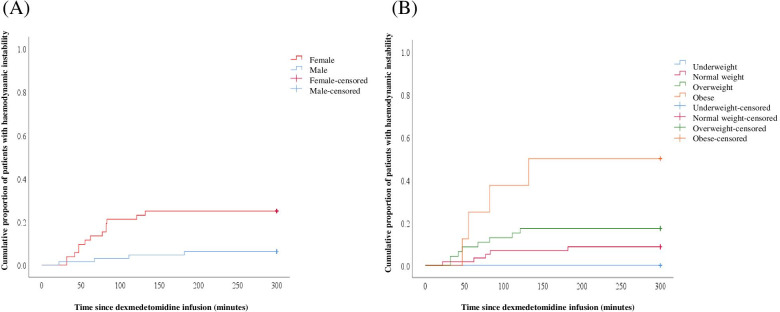
Cumulative incidence of dexmedetomidine-induced hemodynamic instability in females and males (**A**) and in stratified groups according to body mass index (**B**)

We performed additional analysis, using the survival curve, followed by log-rank test and post-hoc analysis, with stratified patients’ groups according to the sex and BMI (Fig. 3). The cumulative incidence of DMED-induced hemodynamic instability was higher in females than in males (*P* = 0.004). When the analysis was done after stratifying the patients by BMI, the cumulative incidence of DMED-induced hemodynamic instability was significantly higher in obese patients than in normal-weight or overweight patients (*P* = 0.001 and *P* = 0.044, respectively).

## Discussions

The current study characterizes hemodynamic instability in patients sedated with DMED during surgery under regional anesthesia. Even though the hemodynamic effects of DMED, including hypotension and bradycardia, are well known [5, 12–14], there have been few related clinical practice investigations. In the current study, the prevalence of DMED-induced hemodynamic instability in regional anesthesia practice was found to be 14.7%, and the event occurred at a median time of 67.0 (IQR 37.4–96.6) minutes after initiating DMED administration. Similar results were reported in a study that followed-up patients for six hours. It was found that DMED induced a maximal decrease in blood pressure at 60 min after administration of a loading dose [5]. Remarkably, most patients (16/17) in the unstable group in our study experienced the hemodynamic instability during their stay at the PACU, regardless of the infusion time of DMED. Only one patient experienced hypotension during surgery while receiving DMED. The authors think that the release of vasodilating substances which had been accumulated in the surgical arm with pneumatic tourniquet might affect the development of hypotensive event not during the surgery but after the surgery. Moreover, the hypotensive effect of DMED in the unstable group was sustained until discharge from the PACU. This phenomenon suggests that patients administered DMED during surgical procedures should be monitored closely not only during the surgery but also during the recovery phase.

The hemodynamic change following DMED infusion, which was found to be dose-dependent and typically biphasic, is characterized by transient hypertension and reflex bradycardia, followed by hypotension [4, 5]. At the initial loading phase, as the plasma level of DMED abruptly increases, hypertension and reflex bradycardia often occur as a result of peripheral vasoconstriction through postsynaptic α2-adrenoreceptor activation in the vascular smooth muscles. After the initial loading, when the plasma concentration of DMED decreases, activation of the postsynaptic α2-adrenoreceptors in the vascular endothelial cells causes peripheral vasodilatation. Simultaneously, activation of the presynaptic α2-adrenoreceptors also contributes to inducing hypotension during this phase [12–14]. Transient hypertensive episode following DMED loading can be eliminated by decreasing the loading dose or elongating the time to peak plasma concentration [5, 15]. In the current study, only six patients in the stable group presented a significant hypertensive episode after infusion of 1 µg/kg of DMED over ten minutes. Among them, two had a history of concomitant hypertension and diabetes mellitus, two had a history of diabetes mellitus for over ten years, and one had a history of hypertension. Possible predictors for a hypertensive episode following DMED loading and a new dosing scheme for susceptible populations would be investigated in future studies.

Ice et al. [9] suggested that older age was an independent risk factor for DMED-induced hemodynamic instability in ICU patients, despite the influence of age on DMED pharmacokinetics not being conclusively proven [16, 17]. The current study expanded the potential risk factors to include sex and BMI. Female sex and obesity were found to be independent risk factors for DMED-induced hemodynamic instability, while older age (> 65 years) did not affect it. Because obese patients generally have more fat tissue, the proportion of the lean body tissue to the total body weight is lower in this population. In addition, the blood flow is distributed mainly to the vessel-rich or lean body tissues (brain, muscle, heart, etc.), as fat tissue is normally poorly perfused. Consequently, dosing based on total body weight is prone to overdose obese patients and results in a higher peak plasma concentration of the drug than lean subjects [18, 19]. DMED, a highly lipophilic drug, has a large volume of distribution. It can rapidly distribute to fatty tissues and pass the blood–brain barrier into the brain [4]. It is well-known that the distribution of the lipophilic drugs to fat or lean body tissues could alter their pharmacokinetics. In several studies investigating the pharmacokinetics of DMED in obese patients, peak plasma concentration was significantly higher in obese patients, even with a higher volume of distribution, than observed in normal-weight patients when dosed by total body weight [19–21]. Based on the current study’s results, these pharmacokinetic differences might contribute to the more pronounced hypotension and other cardiovascular effects of DMED in obese patients. Even though females generally have a higher proportion of fat tissue to total body weight, the precise mechanisms explaining why the DMED-induced hemodynamic instability develops more often in females should be investigated in future.

The study has some limitations. First, inter-individual variability was not controlled in the study. Hypoalbuminemia and lower cardiac output were suggested to induce prolonged DMED effects in critically ill patients [17]. DMED has a high protein binding capability, with 94% of it bound to albumin and α1-glycoprotein. Hypoalbuminemia can thus be expected to affect the drug’s pharmacokinetics [4, 17]. However, all enrolled participants in the current study were healthy patients with ASA/PS I-III. Subjects in critical medical conditions were excluded from the study. Furthermore, several factors that might induce inter-individual variabilities, such as ethnicity and genetic polymorphism, have been suggested [21]. Yet, there are no clinical recommendations to guide dosing regimens by population type. Second, indirect measurement of blood pressure in the current study might be prone to error in identifying the DMED-induced hemodynamic instability. Third, we did not investigate whether the DMED-induced hemodynamic instability could negatively affect perioperative outcomes. Mathis MR et al. has shown that hypotension did not increase the risk of postoperative organ injury in patients with low risk [22]. The clinical significance of short and long-term perioperative cardiovascular or neurologic outcomes even in high risk patients will be clarified in future studies. Finally, this study is limited due to the retrospective design and the small number of participants. Further investigation in a larger study population would be needed.

## Conclusions

In conclusion, this retrospective cohort study found a prevalence of DMED-induced hemodynamic instability of 14.7% in patients administered with intravenous DMED at a loading dose of 1 μg/kg, followed by 0.2–0.6 μg/kg/hr for sedation during orthopedic upper limb surgery under BPB. Female sex and obesity were found to be associated with a higher probability of developing DMED-induced hemodynamic instability. These findings suggest that the administered dose of DMED may be diminished to prevent hypotensive risk in female and obese patients.

## Data Availability

The data analyzed during the current study are available from the corresponding author on reasonable request.

## Abbreviations

DMED: Dexmedetomidine
ICU: Intensive care unit
BPB: Brachial plexus block
ASA PS: American Society of Anesthesiologist Physical Status
BMI: Body mass index
PACU: Postanesthesia care unit
SBP: Systolic blood pressure
RM ANOVA: Repeated measures analysis of variance
OR: Odds ratio
CI: Confidence interval
